# Investigating hydrocephalus using Multiple-network Poroelastic Theory

**DOI:** 10.1186/2045-8118-12-S1-P54

**Published:** 2015-09-18

**Authors:** John Christopher Vardakis, BJ Tully, L Guo, Y Ventikos, D Chou

**Affiliations:** 1 University College London, United Kingdom; 2 First Light Fusion Ltd., UK; 3 Institute of Biomedical Engineering and Department of Engineering Science, University of Oxford, Oxford, UK

## Introduction

The function of the brain depends on the transport of a multitude of fluids, namely blood, cerebrospinal fluid, interstitial fluid and intracellular fluid. Our ability model these intertwined fluid transport processes within brain tissue in an anatomically accurate and patient-specific manner is of ever-increasing significance, especially since integrative systems possess numerous interactions with the external world which either directly or indirectly affect brain function and homeostasis.

## Methods

The current state of knowledge about hydrocephalus (HCP), and more broadly integrative cerebral dynamics and its associated constitutive requirements, advocates that poroelastic theory provides a suitable framework to better understand such a disease. Multiple-network Poroelastic Theory is used to develop a novel spatio-temporal model of tissue displacement and fluid regulation in varying scales within the cerebral environment. The system of equations is discretized in a variety of formats, and in all three spatial dimensions. Both obstructive (mild and severe aqueductal stenosis, 4th ventricle outlet obstruction) and communicating hydrocephalus was investigated in a variety of settings, and accompanied by surgical techniques such as Endoscopic Third and Fourth Ventriculostomy (ETV and EFV). Aquaporin-4 swelling characteristics have also been incorporated into this MPET through the use of simple, functional relationships.

## Results

Ventriculomegaly, CSF/ISF pressure, wall shear stress and pressure difference between lateral and fourth ventricles increased with applied stenosis, and subsequently dropped to nominal levels with the application of ETV. The greatest reversal of the effects of atresia of the 4th ventricle comes by opting for ETV rather than the more complicated procedure of EFV. Periventricular swelling can also be observed at various stages of HCP development, in both obstructive and communicating HCP simulations.

## Conclusions

This work presents an assessment of the impact of aqueductal stenosis and 4th ventricle outlet obstruction, along with the applications of ETV and EFV on an anatomically accurate representation of the cerebroventricular system. Theoretical adaptations to communicating HCP were also investigated. Evolution of the MPET model can lead to a level of complexity that could allow for an experimentally guided exploration of areas that would otherwise prove too intricate and intertwined under conventional settings.

